# Scrambled body differentiates body part ownership from the full body illusion

**DOI:** 10.1038/s41598-020-62121-9

**Published:** 2020-03-24

**Authors:** Ryota Kondo, Yamato Tani, Maki Sugimoto, Masahiko Inami, Michiteru Kitazaki

**Affiliations:** 1 Department of Computer Science and Engineering, Toyohashi University of Technology, 1-1 Hibarigaoka, Tempaku, Toyohashi, Aichi 441-8580 Japan; 2 Department of Information and Computer Science, Keio University, 3-14-1 Hiyoshi, Kohoku-ku, Yokohama, Kanagawa 223-8522 Japan; 3 Research Center for Advanced Science and Technology, The University of Tokyo, 7-3-1 Hongo, Bunkyo-ku, Tokyo, 113-0033 Japan

**Keywords:** Human behaviour, Perception

## Abstract

Illusory body ownership can be induced in a body part or a full body by visual-motor synchronisation. A previous study indicated that an invisible full body illusion can be induced by the synchronous movement of only the hands and feet. The difference between body part ownership and the full body illusion has not been explained in detail because there is no method for separating these two illusions. To develop a method to do so, we scrambled or randomised the positions of the hands and feet and compared it with the normal layout stimulus by manipulating visual-motor synchronisation. In Experiment 1, participants observed the stimuli from a third-person perspective, and the questionnaire results showed that the scrambled body stimulus induced only body part ownership, while the normal layout stimulus induced both body part ownership and full body ownership when the stimuli were synchronous with participants’ actions. In Experiment 2, we found similar results as with the first-person perspective stimuli in a questionnaire. We did not find significant skin conductance response difference between any conditions in either Experiment 2 or 3. These results suggest that a spatial relationship is necessary for the full body illusion, but not for body part ownership.

## Introduction

### Body part ownership

Humans can have illusory ownership of part of a fake body. In the rubber hand illusion, an experimenter simultaneously strokes a rubber hand and the participant’s hand with a brush. The participants feel as if the rubber hand is their own hands when they observe only the rubber hand^1,2^. When the rubber hand illusion is induced, the proprioceptive sensation of the hand drifts toward the rubber hand. However, Rohde, Di Luca, and Ernst (2011)^3^ suggest that proprioceptive drift does not correlate with the illusion. The rubber hand illusion does not occur when the rubber hand is rotated 90 degrees or replaced with a wood-stick, suggesting that the rubber hand illusion is not based on purely bottom-up processes, but also modulated by top-down influences originating from the appearance of one’s own body^2^. Ide (2013)^4^ showed that the anatomical plausibility of hand posture affects the rubber-hand illusion; For the left hand, the illusion was stronger when the rubber hand was placed at 0°, 45°, 90° (easy to mimic with the actual left hand), and 315° than 180°, 225°, 270° (difficult to mimic with the actual left hand by the anatomical constraint). Thus, the incongruence in bottom-up visual and proprioceptive signals could explain why the rubber-hand illusion is eliminated or decreased with the rotated rubber hand. The similar finding has been reported in the fMRI study^5^; When the rubber hand is put on the same posture as real hand, the illusion is induced and the premotor cortex showed stronger activation than when the rubber hand is rotated 180 degrees. In those studies, the illusion is induced by visual-tactile synchronicity. The virtual hand illusion^6^ and the moving rubber hand illusion^7,8^ have both been reported using visual-motor synchronicity. In the virtual hand illusion, a virtual arm is presented on a screen synchronised with the participant’s hand movements. The participants then feel as if the virtual arms is their own.

### Full body illusion (FBI)

The rubber hand illusion induces the illusory ownership of body parts, but does not induce that of a whole body. The illusory ownership of a full body has been investigated (full body illusion). In the full body illusion, participants feel as if a mannequin^9^ or a full body avatar^10–12^ is their own body due to visual-motor or visual-tactile synchronicity. The full body illusion using a mannequin is induced by stroking the mannequin and the participant’s body at the same time, while the participant observes the stroked abdomen from the position of the mannequin’s head through a head-mounted display (HMD). In a full body illusion of a virtual reality avatar, the avatar moves synchronously with a participant’s movements^10^, or visual-tactile stimuli are presented using virtual balls and vibrations^12^. The full body illusion is stronger and more likely to be induced through a visual-tactile experience from the first-person perspective than the third-person perspective^11,12^. In the first-person perspective, participants observed the virtual room and the virtual body from the point of view of the virtual body’s eyes. In the third-person perspective, the position of participants (the point of view) was horizontally shifted 1 m^11^ or 40 cm^12^ away to the right of the virtual body; Participants could see the virtual body when they turn their head left. When participants observe a fake body from the front (facing each other), the full body illusion does not occur^13,14^. On the other hands, the full body illusion can be induced when participants observe a body from the third-person perspective under certain conditions^15–17^. Participants observe a fake body from behind using an HMD while an experimenter simultaneously strokes the back of the fake body and that of the participant. When the visual and tactile strokes are synchronised, participants experience the full body illusion with the fake body, and their self-localisation drifts towards the fake body. In these studies, participants observe the fake body from behind. However, the self-localisation effect has not been replicated in some studies^18–20^, and thus it is controversial. The full body illusion from the third person perspective is a kind of self-recognition similar to recognising oneself in a mirror according to Petkova *et al*.^13^ and Ehrsson (2012)^21^. Also, Maseli and Slater (2013)^12^ discussed that the difference in the illusion strength between the first-person perspective and the third-person perspective can be explained by their theory.

### Invisible body illusion

Illusory body part ownership and the full body illusion can be adapted to create an invisible body illusion. The invisible body illusion is induced by visual-tactile synchronicity (invisible hand^22^, invisible full body^23^, and invisible small or large body^24^) or visual-motor synchronicity^25^. In the visual-tactile method, an experimenter strokes a participant’s body with a brush and the participant observes the corresponding movement of the brush in empty space through an HMD. In the visual-motor method, participants observe virtual gloves and socks that move synchronously with their own movements. They then feel as if the space between the gloves and socks is their own body, and the invisible body is perceived by interpolating the body parts. This result suggests that the spatial relationship of body parts and the synchronous movement of the hands and feet are important to induce the full body illusion.

### Body part ownership versus the full body illusion

Several studies have discussed the difference between body part ownership and the full body illusion. Blanke and Metzinger (2009)^26^ and Blanke (2012)^27^ claim that body part ownership and the full body illusion are fundamentally different in terms of bodily self-consciousness. The bilateral premotor cortex, intraparietal sulcus, and the cerebellum are associated with the rubber hand illusion^5,28^. Activity in the right posterior insula and right frontal operculum correlates with the proprioceptive drifts in the rubber hand illusion, although the activation in these areas does not increase in the synchronous illusion condition compared to the control conditions^29^. Activity in the extrastriate body area reflects the intensity of the rubber hand illusion^30^. On the other hand, activity in the ventral premotor cortex reflects the full body illusion; the full body illusion is only induced by means of visual-tactile stimuli when the body segment connects to a body^31,32^. Even though several studies have investigated the difference between body part ownership and the full body illusion, the difference has not been well understood because an experimental method to separate these two illusions has not yet been developed.

### Aim and hypothesis

**Figure 1:**
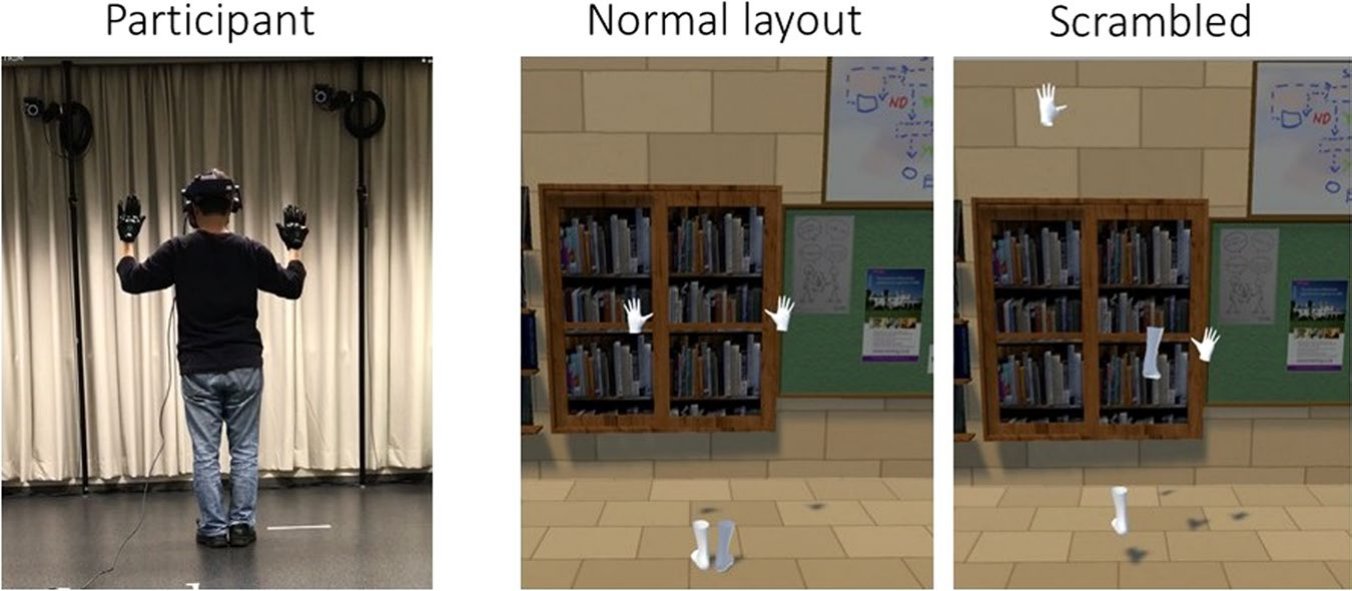
An example of a participant’s posture (left), the corresponding normal layout stimulus (middle), and the scrambled stimulus (right).

In this study, we aimed to develop a method to separate and compare these two illusions. Our previous study suggested that the appropriate spatial relationship of body parts and the synchronous movement of hands and feet are sufficient for the full body illusion^25^. Thus, we hypothesised that the full body illusion requires the spatial relationship of body parts similar to our normal body in addition to visual-motor synchronisation, while body part ownership is induced by means of visual-motor synchronisation without the appropriate spatial relationship. Based on this hypothesis, we developed scrambled stimuli that disrupt the spatial relationship by randomising the positions of body parts from the original/normal body part layout stimulus to induce only body part ownership. Only the gloves and socks were presented both in the normal layout stimulus and the scrambled stimulus as synchronous or asynchronous to the participants’ actions (Fig. 1). We then investigated the ownership of body parts and a full body by comparing the scrambled body stimulus with the normal body part layout stimulus. We hypothesised that participants could have illusory ownership of a full body only when the hands and feet had the normal spatial relationship (normal layout) and were synchronous to their actions, while they could have illusory ownership of hands and feet even from the scrambled stimulus that was synchronous with their actions. In the scrambled stimuli, we did not change the orientation of body parts, and changed only the spatial relationship (position) because it is known that the rotation prevents the body part ownership illusion^2,5^.

In Experiment 1, we measured the body part ownership and full body illusion after the observation of the stimuli using a questionnaire and a self-localization task. Participants observed the gloves and socks from a third-person perspective (behind the stimuli) for the self-localization task. We expected that if they felt as if the invisible body in the front were their own full body, they might move toward the invisible body (further forward from the initial position).

**Figure 2:**
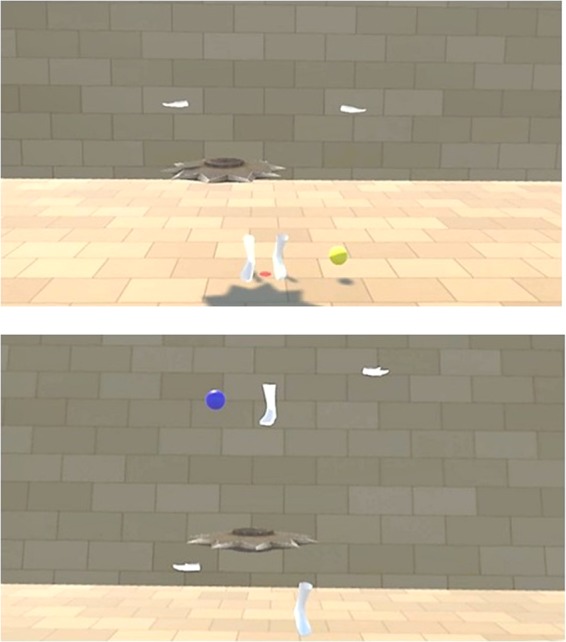
A virtual wheel cutter and virtual gloves and socks in a virtual mirror.

In Experiment 2, participants observed the gloves and socks from the first-person perspective to examine whether the body ownership illusion was stronger from the first-person perspective. A questionnaire and skin conductance response (SCR) were used to investigate the body part ownership and full body illusion. A virtual wheel cutter cut an empty space between the gloves and socks (Fig. 2). We expected that if the full body illusion was induced in the empty space, the SCR would increase when the cutter cut the empty space.

In Experiment 3, we measured the SCR to the sudden appearance of the virtual wheel cutter, similarly to Experiment 2. However, the timings of the threatening events were random to prevent participants’ anticipation.

All experiments were conducted in within-subject design; all participants (16 naïve participants for Experiment 1, 16 naïve participants for Experiment 2, and 20 naïve participants for Experiment 3) experienced all four conditions (a combination of normal layout and scrambled bodies, and synchronous and asynchronous movements).

## Results

### Experiment 1

#### Questionnaire

We conducted the Shapiro-Wilk test to check the normality of the results of the questionnaire, and found that the data could not be assumed to have come from a normally distributed population. Therefore, we applied the Wilcoxon signed-rank test to analyse the results of the questionnaire (body: normal layout, scrambled; synchronisation: synchronous, asynchronous; *N* = 16). *P*-values were corrected by the Bonferroni method.

**Figure 3:**
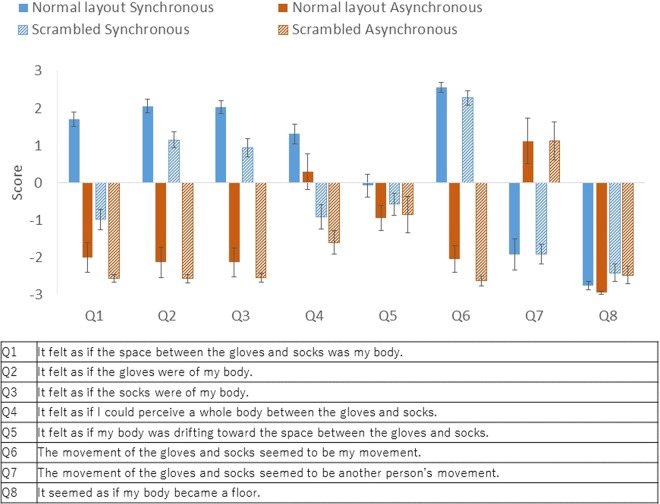
Results of the questionnaire in Experiment 1. Error bars indicate SE.

The results of the questionnaire (scored on a seven-point Likert scale from −3 to 3) are summarised in Fig. 3. The participants were more likely to feel that the space between the gloves and socks was their own body in the normal layout body condition than in the scrambled condition only when the stimuli moved synchronously (Q1: *z* = 3.53, original *p* < 0.001, adjusted *p* < 0.001, effect size *r* = 0.88). They were more likely to feel that the space between the gloves and socks was their own body in the synchronous condition than in the asynchronous condition in both the normal layout condition (Q1: *z* = 3.47, original *p* < 0.001, adjusted *p* < 0.001, *r* = 0.87) and the scrambled condition (Q1: *z* = 3.31, original *p* < 0.001, adjusted *p* < 0.001, *r* = 0.83). Data of all participants are provided as Supplementary Material.

They were more likely to feel that the gloves (Q2) or socks (Q3) were part of their own bodies in the normal layout condition than in the scrambled condition when the stimuli moved synchronously (Q2: *z* = 3.11, original *p* < 0.001,adjusted *p* = 0.0051, *r* = 0.78, Q3: *z* = 3.33, original *p* < 0.001, adjusted *p* < 0.001, *r* = 0.83), and were more likely to feel that the gloves (Q2) or socks (Q3) were part of their own bodies in the synchronous condition than in the asynchronous condition in both the normal layout condition (Q2: *z* = 3.42, original *p* < 0.001, adjusted *p* < 0.001, *r* = 0.85; Q3: *z* = 3.47, original *p* < 0.001, adjusted *p* < 0.001, *r* = 0.87) and the scrambled condition (Q2: *z* = 3.54, original *p* < 0.001, adjusted *p* < 0.001, *r* = 0.89; Q3: *z* = 3.53, original *p* < 0.001, adjusted *p* < 0.001, *r* = 0.88).

They were also more likely to feel that there was a whole body (invisible body) between the gloves and socks in the normal layout condition than in the scrambled condition in both the synchronous condition (Q4: *z* = 3.45, original *p* < 0.001, adjusted *p* < 0.001, *r* = 0.86) and the asynchronous condition (Q4: *z* = 3.22, original *p* < 0.001, adjusted *p* = 0.0022, *r* = 0.81).

There was no significant difference between the synchronous condition and the asynchronous condition with either the normal layout body (Q4: *z* = 1.87, original *p* = 0.068, adjusted *p* = 0.41, *r* = 0.47) or the scrambled body in the perception of an invisible body (Q4: *z* = 1.72, original *p* = 0.093, adjusted *p* = 0.56, *r* = 0.43).

There was no significant difference between the body condition and the synchronisation conditions on the subjective drift of self-location (Q5 Normal Layout Synchronous vs. Scrambled Synchronous: *z* = 1.09, original *p* = 0.29, adjusted *p* = 1.00, *r* = 0.27). There was no significant difference between the synchronous condition and the asynchronous condition (Q5 Normal Layout Synchronous vs. Normal Layout Asynchronous: *z* = 1.86, original *p* = 0.066, adjusted *p* = 0.39, *r* = 0.46; Scrambled Synchronous vs. Scrambled Asynchronous: *z* = 0.79, original *p* = 0.46, adjusted *p* = 1.00, *r* = 0.20).

The participants were more likely to feel that the movements of the gloves and socks were their own movements in the synchronous condition than in the asynchronous condition in both the normal layout condition (Q6: *z* = 3.54, original *p* < 0.001, adjusted *p* < 0.001, *r* = 0.88) and the scrambled condition (Q6: *z* = 3.53, original *p* < 0.001, adjusted *p* < 0.001, *r* = 0.88), and were more likely to feel that the movements of the gloves and socks were someone else’s movements in the asynchronous condition than in the synchronous condition in both the normal layout condition (Q7: *z* = −2.68, original *p* < 0.001, adjusted *p* < 0.001, *r* = 0.67) and the scrambled condition (Q7: *z* = 3.01, original *p* = 0.0013, adjusted *p* = 0.0079, *r* = 0.75).

The eighth question (‘It seemed as if my body became a floor’) was a control question to test the reliability of participants’ judgments. No significant difference was found for the control question.

#### Self-localisation drift

One participant was excluded from the data of the self-localisation task after colliding with a wall during the task. We measured the distance between the initial position and the estimated position in the self-localisation task and subtracted the value of the control trial from that of the experimental trials to control participants’ individual differences. We conducted a two-way repeated-measure ANOVA to analyse the results of the self-localisation task (Body: normal layout, scrambled; Synchronisation: synchronous, asynchronous; *N* = 15).

**Figure 4:**
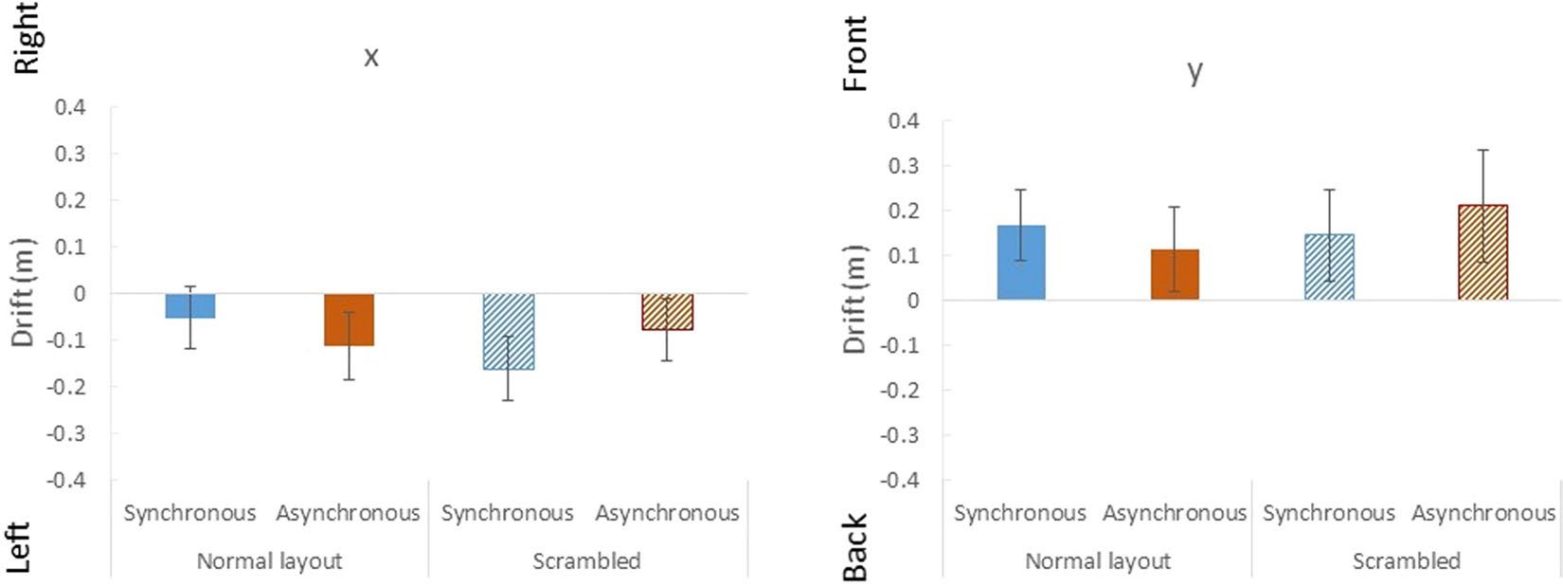
Results of self-localisation drift in Experiment 1. Error bars indicate SE.

There was no significant difference for either the *x* (left-right) axis (No main effect of the body: *F* (1, 14) = 0.35, *p* = 0.56, *η*_*p*_^2^ = 0.024; No main effect of synchronisation: *F* (1, 14) = 0.039, *p* = 0.85, *η*_*p*_^2^ = 0.0027; No interaction: *F* (1, 14) = 2.39, *p* = 0.14, *η*_*p*_^2^ = 0.15) or the *y* (forward-backward) axis (No main effect of the body: *F* (1, 14) = 0.27, *p* = 0.61, *η*_*p*_^2^ = 0.019; No main effect of synchronisation: *F* (1, 14) = 0.0039, *p* = 0.95, *η*_*p*_^2^ = 0.0003; No interaction: *F* (1, 14) = 0.57, *p* = 0.46, *η*_*p*_^2^ = 0.039), as shown in Fig. 4. Thus, we did not find any drift in the proprioceptive self-location.

### Experiment 2

Eighteen participants performed the experiment, but two were excluded from the questionnaire and skin conductance response (SCR) because one did not look down when the cutter appeared and the SCR data was not recorded, while the other also did not look down when the cutter appeared and the Vive Tracker was removed during the ball task.

We conducted the Shapiro-Wilk test to check the normality of the results of the questionnaire and SCR, and found that the data could not be assumed to have come from a normally distributed population. Therefore, we conducted a Wilcoxon signed-rank test to analyse the results of the questionnaire and SCR (body: normal layout, scrambled; synchronisation: synchronous, asynchronous; *N* = 16). *P*-values were corrected by the Bonferroni method.

#### Questionnaire

**Figure 5:**
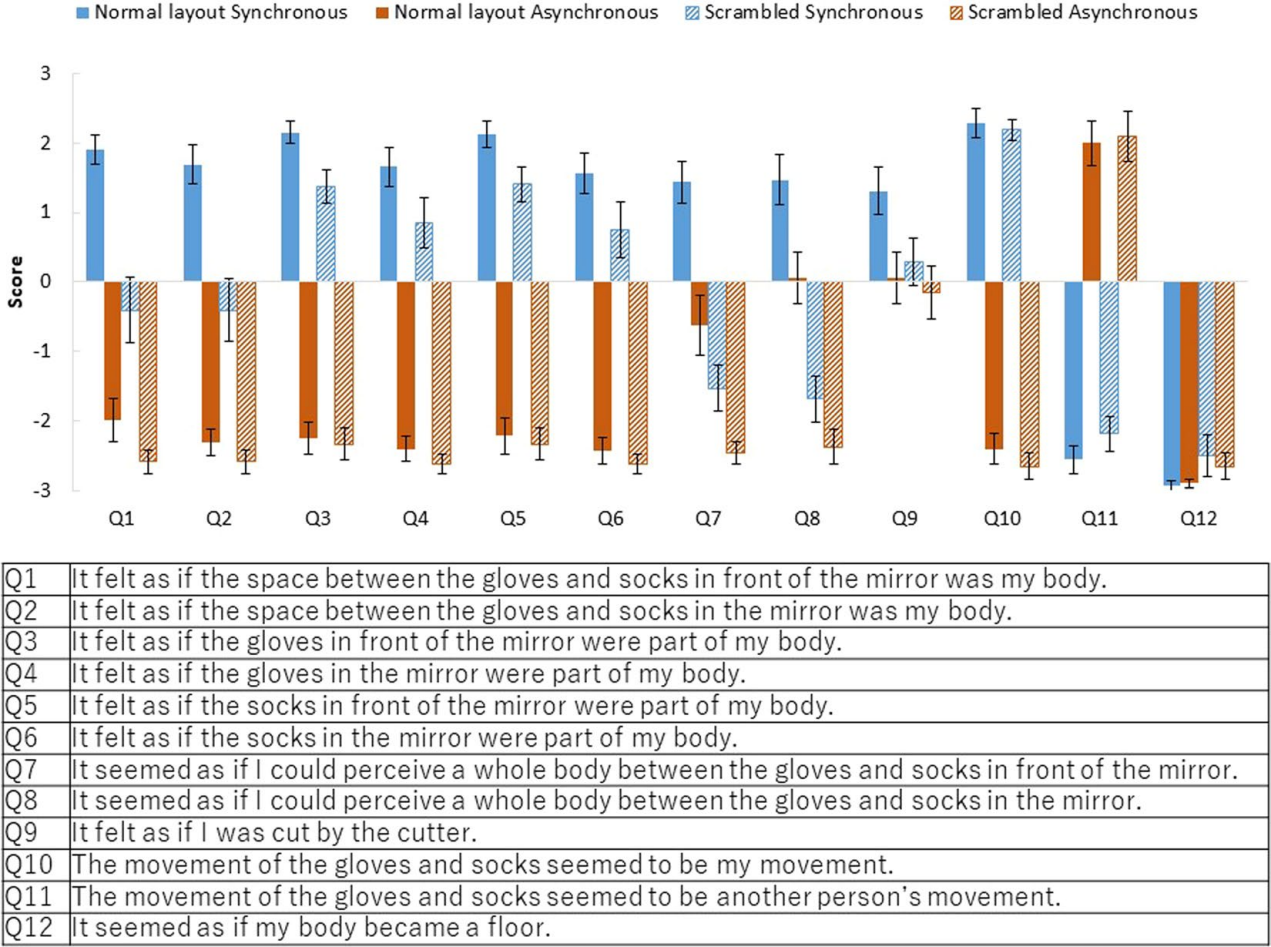
Results of the questionnaire in Experiment 2. Error bars indicate SE.

The results of the questionnaire (seven-point Likert scale from −3 to 3) are summarised in Fig. 5. Participants were more likely to feel that the space between the gloves and socks was their own body in the synchronous condition than in the asynchronous condition both in the normal layout condition (Q1 (in front of mirror): *z* = 3.42, original *p* < 0.001, adjusted *p* = <0.001, effect size *r* = 0.85; Q2 (in mirror): *z* = 3.52, original *p* < 0.001, adjusted *p* = <0.001, *r* = 0.88) and scrambled conditions (Q1: *z* = 2.98, original *p* < 0.001, adjusted *p* = 0.0059, effect size *r* = 0.75; Q2: *z* = 3.18, original *p* < 0.001, adjusted *p* = 0.0015, *r* = 0.80); the effect was larger in the normal layout condition. The score was higher in the normal layout condition than in the scrambled condition both in the synchronous condition (Q1: *z* = 3.30, original *p* < 0.001, adjusted *p* = <0.001, effect size *r* = 0.83; Q2: *z* = 3.19, original *p* < 0.001, adjusted *p* = 0.0015, *r* = 0.80) and the asynchronous condition (Q1: *z* = 2.55, original *p* = 0.0078, adjusted *p* = 0.047, effect size *r* = 0.64; but not significant for Q2 *z* = 2.26, original *p* = 0.031, adjusted *p* = 0.188, effect size *r* = 0.57); the effect was much larger in the synchronous condition. Thus, the participants felt as if the space between the gloves and socks was their own body only in the synchronous and normal layout condition (average scores Q1: 1.91, Q2: 1.69).

They were more likely to feel that the gloves (Q3 (in real space), Q4 (in mirror)) or socks (Q5 (in real space), Q6 (in mirror)) were part of their own bodies in the synchronous condition than in the asynchronous condition in both the normal layout condition (Q3: *z* = 3.54, original *p* < 0.001, adjusted *p* = <0.001, *r* = 0.88; Q4: *z* = 3.52, original *p* < 0.001, adjusted *p* = <0.001, *r* = 0.88; Q5: *z* = 3.52, original *p* < 0.001, adjusted *p* = <0.001, *r* = 0.88; Q6: *z* = 3.52, original *p* < 0.001, adjusted *p* = <0.001, *r* = 0.88) and the scrambled condition (Q3: *z* = 3.52, original *p* < 0.001, adjusted *p* = <0.001, *r* = 0.88; Q4: *z* = 3.52, original *p* < 0.001, adjusted *p* = <0.001, *r* = 0.88; Q5: *z* = 3.52, original *p* < 0.001, adjusted *p* = <0.001, *r* = 0.88; Q6: *z* = 3.52, original *p* < 0.001, adjusted *p* = <0.001, *r* = 0.88). The score was higher in the normal layout condition than in the scrambled condition in the synchronous condition (Q3: *z* = 2.84, original *p* = 0.0035, adjusted *p* = 0.021, *r* = 0.71; Q4: *z* = 2.73, original *p* = 0.0048, adjusted *p* = 0.029, *r* = 0.68; Q5: *z* = 2.96, original *p* < 0.001, adjusted *p* = 0.0059, *r* = 0.74).

They were more likely to feel that there was a whole body (invisible body) between the gloves and socks in front of the mirror in the synchronous condition than in the asynchronous condition both in the normal layout condition (Q7: *z* = 2.90, original *p* = 0.0018, adjusted *p* = 0.011, effect size *r* = 0.73) and scrambled condition (Q7: *z* = 2.53, original *p* = 0.0078, adjusted *p* = 0.047, effect size *r* = 0.63); the effect was larger in the normal layout condition. The score was higher in the normal layout condition than in the scrambled condition both in the synchronous condition (Q7: *z* = 3.41, original *p* < 0.001, adjusted *p* = <0.001, effect size *r* = 0.85) and the asynchronous condition (Q7: *z* = 2.95, original *p* = 0.0015, adjusted *p* = 0.0087, effect size *r* = 0.74). Only in the normal layout condition were participants more likely to feel that there was a whole body (invisible body) between the gloves and socks in the mirror in the synchronous condition than in the asynchronous condition (Q8: *z* = 2.75, original *p* = 0.0042, adjusted *p* = 0.025, effect size *r* = 0.69). The score was higher in the normal layout condition than in the scrambled condition both in the synchronous condition (Q8: *z* = 3.53, original *p* < 0.001, adjusted *p* = <0.001, effect size *r* = 0.88) and the asynchronous condition (Q8: *z* = 3.25, original *p* < 0.001, adjusted *p* = 0.0015, effect size *r* = 0.81). Thus, the participants surely felt as if there was an invisible body between the gloves and socks only in the synchronous and normal layout conditions (average scores Q7 (in front of mirror): 1.44, Q8 (in mirror): 1.47).

They were more likely to feel as if they would be cut by the cutter in the synchronous condition than in the asynchronous condition only in the normal layout condition (Q9: *z* = 2.56, original *p* = 0.0078, adjusted *p* = 0.047, effect size *r* = 0.64), and it was not significant in the scrambled conditions (Q9: *z* = 0.83, original *p* = 0.43, adjusted *p* = 1.00, effect size *r* = 0.21). Thus, they felt as if they would be cut by the cutter when the full body illusion was induced rather than when only body part ownership occurred.

The participants were more likely to feel that the movements of the gloves and socks were their own movements in the synchronous condition than in the asynchronous condition in both the normal layout condition (Q10: *z* = 3.54, original *p* < 0.001, adjusted *p* < 0.001, *r* = 0.88) and scrambled conditions (Q10: *z* = 3.54, original *p* < 0.001, adjusted *p* < 0.001, *r* = 0.89). They were more likely to feel that the movements of the gloves and socks were someone else’s movements in the asynchronous condition than in the synchronous condition in both the normal layout condition (Q11: *z* = −3.54, original *p* < 0.001, adjusted *p* < 0.001, *r* = 0.89) and scrambled conditions (Q11: *z* = −3.54, original *p* < 0.001, adjusted *p* < 0.001, *r* = 0.88).

The twelfth question (‘It seemed as if my body became a floor.’) was a control question to test the reliability of participants’ judgments. No significant difference was found on the control question.

#### Skin conductance response

**Figure 6:**
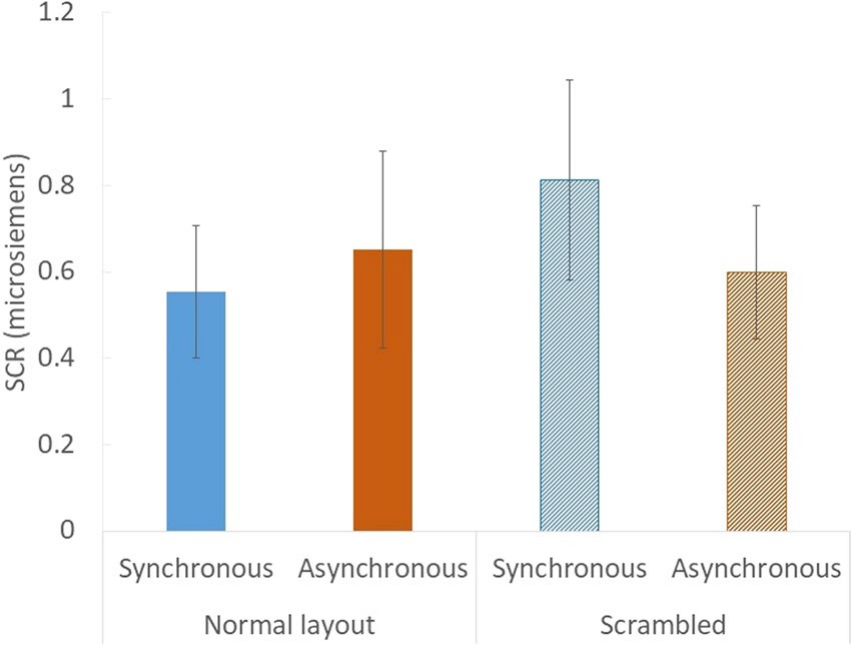
Results of the SCR in Experiment 2. Error bars indicate SE.

The magnitude of the SCR was calculated as the difference between the maximum value and the minimum value of the skin conductance during the 0–5 s after the appearance of the cutter; this procedure was adopted from previous studies^9,13,22,23^. We expected that the magnitude of the SCR would be larger when the full body illusion was induced than when only body part ownership occurred. However, there was no significant difference between the synchronous and asynchronous condition in the normal layout condition (*z* = −0.52, original *p* = 0.63, adjusted *p* = 1.00, *r* = 0.13), or between the normal layout condition and the scrambled condition in the synchronous condition (*z* = −0.41, original *p* = 0.71, adjusted *p* = 1.00, *r* = 0.10), as shown in Fig. 6.

However, there is also a potential methodological concern. Participants were asked to remain standing in a T-pose (standing straight, holding their arms out horizontally) and continue looking down until the end of the trial when the word ‘T-Pose’ appeared in a notification on the HMD. Ten seconds after the T-pose, the cutter appeared and cut the empty space. The skin conductance tended to increase just after the ‘T-Pose’ notification appeared. Therefore, the notification might have evoked the SCR, inhibiting the responses for the cutting threat. In Experiment 3, we removed this anticipating procedure, and presented the cutter three times at random timings during participants’ movements.

### Experiment 3

**Figure 7:**
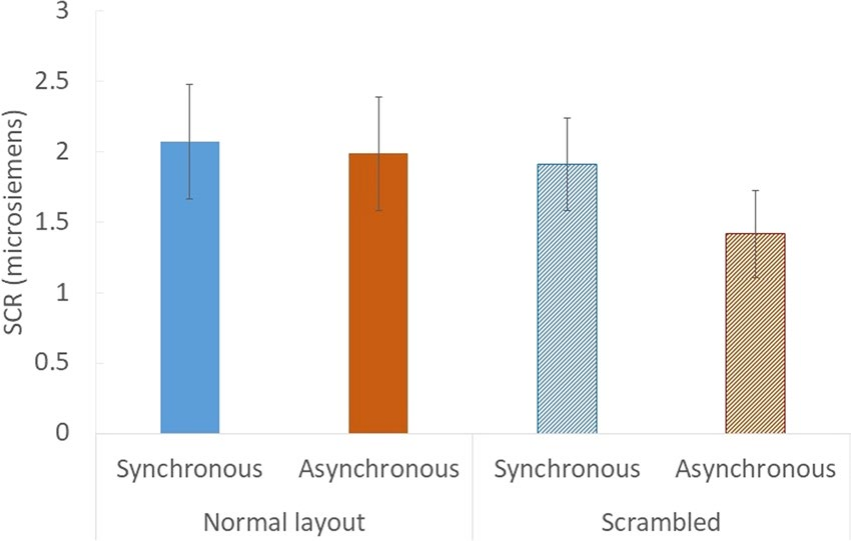
Results of the SCR in Experiment 3. Error bars indicate SE.

Twenty participants performed the experiment and we measured the SCR without the questionnaire. None of them experienced the other experiments. The magnitude of the SCR was calculated as the difference between the maximum and minimum value of the skin conductance during the 0–5 s after the appearance of the cutter. We conducted the Shapiro-Wilk test to check the normality of the results of the SCR, and found that the data could not be assumed to have come from a normally distributed population. Therefore, we conducted a Wilcoxon signed-rank test to analyse the results of the SCR. We expected that the magnitude of the SCR would be larger when the full body illusion was induced than when only body part ownership occurred. However, there was no significant difference between the synchronous and asynchronous conditions in the normal layout condition (*z* = 0.26, original *p* = 0.81, adjusted *p* = 1.00, *r* = 0.058), or between the normal layout condition and the scrambled condition in the synchronous condition (*z* = 0.11, original *p* = 0.93, adjusted *p* = 1.00, *r* = 0.025), as shown in Fig. 7.

## Discussion

In this study, we developed techniques to investigate the scrambled body and compared body part ownership and the full body illusion. We found that participants felt as if the space between the gloves and socks was their own bodies only when the normal layout stimuli were synchronous with their movements both in the third- and first-person perspective (Experiment 1 Q1, Experiment 2 Q1, Q2: Full body illusion). They felt as if the gloves or socks were part of their own bodies in both body conditions (Experiment 1 Q2, Q3; Experiment 2 Q3, Q4, Q5, Q6: body part ownership), and the feeling was stronger in the normal layout condition than in the scrambled body condition. The invisible whole body was more likely to be perceived by interpolating the hands and feet when the body parts were normally presented than when they were scrambled, and the stimuli were more synchronous than asynchronous with the participants’ actions (Experiment 1 Q4:, Experiment 2 Q7, Q8: invisible body). We did not find self-localisation drift in the questionnaire (Experiment 1 Q5) or in the behavioural task. We did not find significant SCR differences between any conditions by a threatened event in either Experiment 2 or 3.

The results suggest that the spatial relationship between normal body parts is necessary for the full body illusion, but not for body part ownership. Thus, the appropriate spatial relationship of body parts with visual-motor synchronisation is a critical factor for full body ownership.

Body part ownership was induced by the synchronously moving scrambled body parts without full body ownership. Thus, the spatial relationship would not be a necessary condition for body part ownership, while visual-motor synchronisation would be an independent critical factor. However, the spatial relationship improved body part ownership. It is still not clear whether the spatial relationship directly affects body part ownership or if full body ownership increases body part ownership.

We could extract body part ownership from full body ownership because the scrambled body part condition elicited body part ownership without full body ownership. However, whether full body ownership can be induced without body part ownership is an open question. This question is our next research target.

In Experiment 1, participants observed virtual body parts from the third-person perspective (behind the virtual body) because it was difficult for the participants to see the virtual body parts, especially in the scrambled condition, and there was a possibility that the scrambled body parts would be obscured. However, the body ownership illusion might be weaker in the third-person than in the first-person perspective. Several studies found that when participants observed a mannequin from the front, the full body illusion was not induced^13,14^. Petkova *et al*. (2011)^13^ and Ehrsson (2012)^21^ claim that the full body illusion from the third-person perspective is a kind of self-recognition similar to recognising oneself in a mirror. In body part ownership, the rubber hand illusion is weakened when the rubber hand is in a distant position^33,34^. Thus, our method using the third-person perspective might weaken illusory body ownership.

Therefore, participants observed the stimuli from the first-person perspective and observed the stimuli through the virtual mirror in Experiment 2. Illusory body ownership can be induced when a body is presented in a mirror^10,14^; there was no significant difference between the mirror condition and the first-person perspective condition in terms of body ownership^14^. Thus, we used the virtual mirror to present a whole image of the body parts.

We expected participants’ self-localisation drift toward the virtual body only in the normal layout and synchronous condition in Experiment 1 with the third-person perspective. However, there was no significant difference in any of the conditions. This may have been caused by the weakened ownership of the illusory body with the third-person perspective and/or the inclusion of the body parts stimuli. However, some studies report that the feeling of body ownership cannot be measured by proprioceptive drift alone^3,35,36^. Thus, we should consider the results of the self-localisation task separately from the results of the questionnaire. Our negative results partly support the view that the self-localisation task does not measure body ownership, and that it is different from the proprioceptive drift that is used to quantify the rubber hand illusion.

We measured the SCR when the cutter cut the empty space between the gloves and socks in Experiments 2 and 3. We expected that the SCR would increase when the full body illusion was induced; however, there was no significant difference in SCR between any of the conditions, suggesting that the feeling of illusory body ownership of the invisible body created by our method might be too weak to elicit SCR. There is another potential concern regarding the SCR results: The sample size was too small to analyse the SCR (N = 16 in Experiment 2, N = 20 in Experiment 3).

It is the most critical limitation of the present study that our findings rely only on subjective measures (questionnaire). One may argue that participants who experienced all four conditions may simply give higher scores to the stimulus that feels more natural irrespective of the feeling of body ownership. Thus, the normal layout and synchronous condition could be rated higher in most questions. However, we instructed participants to answer the questionnaire appropriately, and the data obtained were quantitative (different between questions). The results for the control questions (Q8 in Experiment 1 and Q12 in Experiment 2) were appropriate. To overcome the limitation of the questionnaire, we need to measure SCR with a larger sample size and a more powerful threatening event. One may argue that the between-subject design could be better than the within-subject design to prevent learning effects, especially when any physiological evidence was not found as in our experiments. It would be better to compare our study with another experiment using a between-subject design in future studies.

## Methods

### Experiment 1

#### Participants

Sixteen male volunteers (mean age 21.44 ± 1.46 (*SD*)) participated in Experiment 1, and were not aware of the hypothesis or naïve to the study. They had healthy vision and physical abilities and provided written informed consent before the experiment. One participant was excluded from the data of the self-localisation task due to a problem. This study was approved by the Ethical Committee on Human-Subject Studies of Toyohashi University of Technology and conformed to the committee’s guidelines and regulations.

#### Apparatus

Experimental stimuli were generated by a computer using a Unity Engine and presented through a head-mounted display (HTC Vive Pro, 1440 × 1600 pixel per eye, 110° diagonal, refresh rate 90 Hz). Participants wore gloves and socks with four motion trackers (VIVE Tracker 2018; spatial resolution: within 1 mm; sampling rate: better than 60 Hz; delay: less than 44 ms).

#### Stimuli and conditions

**Figure 8:**
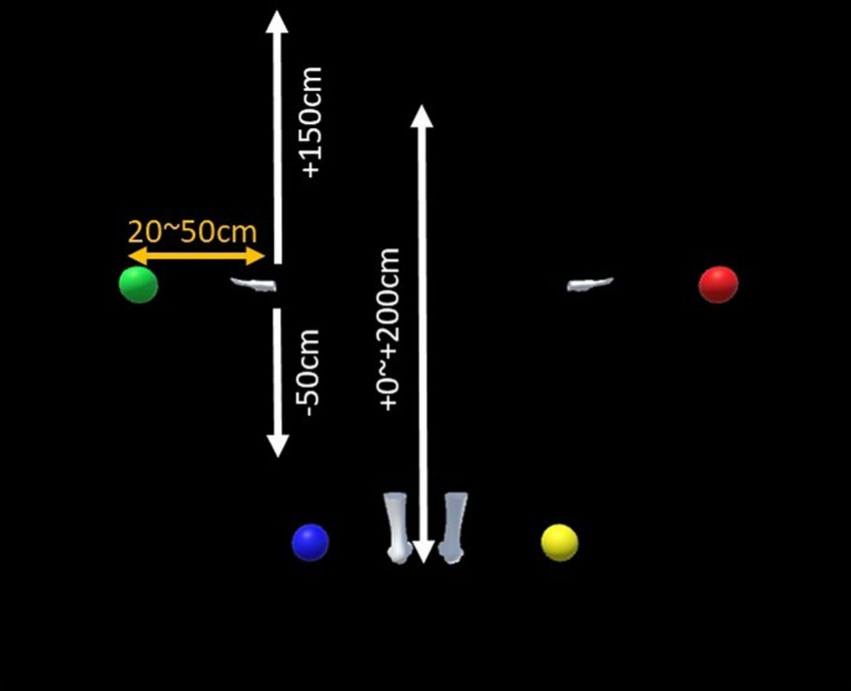
Range of body part positions in the scrambled condition (white), and the range of ball appearance positions (orange).

Virtual gloves and socks were presented in the same positions as the participants’ hands and feet (normal layout condition) or random positions (scrambled condition) in the third-person perspective (Fig. 1); the virtual gloves and socks were 2 m in front of the participants, similar to Kondo *et al*.^25^. The participants always observed the stimuli from behind them (rear view of the body stimuli). In the scrambled body condition, the positions of the gloves and socks were within the ranges of −50 cm to +150 cm and 0 cm to +200 cm from the original vertical positions, respectively (Fig. 8; + indicates up and − down). The gloves and socks moved synchronously or asynchronously with the participant’s hand and foot movements. In the asynchronous condition, another person’s previously recorded motions of the gloves and socks were played irrespective of participant motion; two motions for the normal layout condition and two for the scrambled body condition were prepared and used.

#### Procedure

After participants observed a dark scene for 10 seconds, they observed the gloves and socks from the third-person perspective. A virtual ball appeared on the left or right side of the left-hand or right-hand gloves or socks, respectively, within the range of 20 cm to 50 cm outward from the original position of the participant’s hands and feet as measured at the end of the dark scene (Fig. 8). The virtual ball appeared in one of four colours corresponding to the participant’s right/left hands/feet in a random position. The participants touched the balls with their corresponding hands or feet in random order for 10 minutes. When a ball was touched, it disappeared and reappeared at a different position after 2 seconds (ball task). In the asynchronous condition, the movement of the gloves and socks was a replay of pre-recorded motions. For the ball task, we connected the participants’ hands and feet to invisible gloves and socks (empty spaces) that moved synchronously. The initial position of the invisible body parts was the same as the visible gloves and socks for each trial. After the ball task, the participants performed the self-localisation task^15^. First they were led to an initial position, then moved backwards (2.5, 2.75, 3.0, 3.25, or 3.5 m randomly) and asked to walk to their initial position. All task procedures were conducted without the aid of vision. Finally, they completed a questionnaire comprising eight items on a seven-point Likert scale from −3 (‘I did not feel that at all’) to 3 (‘I felt it very strongly’).

##### Questionnaire

1. It felt as if the space between the gloves and socks was my body.
2. It felt as if the gloves were part of my body.
3. It felt as if the socks were part of my body.
4. It seemed as if I could perceive a whole body between the gloves and socks.
5. It felt as if my body was drifting toward the space between the gloves and socks.
6. The movement of the gloves and socks seemed to be my movement.
7. The movement of the gloves and socks seemed to be another person’s movement.
8. It seemed as if my body became a floor.

The first question concerned full body ownership; the second and third, body part ownership; the fourth, the perception of the invisible body by interpolating the hands and feet; the fifth, the subjective drift of self-location; and the sixth and seventh, the degree of self-agency. The eighth question was a control question to check participants’ unreliable or random responses.

All participants performed eight experimental trials (within-subject design: two body conditions and two synchronisation conditions with two repetitions) in random order and two control trials at the beginning and end of the experiment. Before the trials, the participants touched 10 balls to practise the ball task in the normal layout and synchronous condition.

### Experiment 2

#### Participants

Eighteen naïve volunteers (17 males and 1 female, mean age 22.9 ± 3.04 (SD)) participated in Experiment 2. Two had participated in Experiment 1 four month ago. Two participants were excluded from the data due to procedural problems; the data of sixteen participants were analysed (mean age 23.4 ± 3.72 (SD)).

#### Apparatus

BIOPAC MP 160 and wireless PPG and EDA amplifiers (BN-PPGED) were used to record the skin conductance response (SCR). A wireless transmitter (BN-PPGED) was placed on a participant’s left wrist. Two disposable electrodes (EL507) were pasted to the distal phalanges of their left middle and ring fingers. Two lead cables (BN-EDA-LEAD2) connected the wireless transmitter to the electrodes. SCR data were recorded at 1000 Hz. Other equipment was identical to Experiment 1.

#### Stimuli and conditions

Participants observed the virtual gloves and socks from the first-person perspective and the stimuli through a virtual mirror 2 m in front of them. When they touched the balls, they were asked to look at the balls in front of (not in) the mirror to observe the gloves and socks. A virtual wheel cutter appeared and spun to the left of the participants at the end of each trial. One second after its appearance it passed between the gloves and socks (empty space) in 3 seconds and disappeared. In the scrambled condition, the gloves and socks were in four fixed positions (left gloves: −50 cm; right gloves: +100 cm; left socks: +200 cm; right socks: +50 cm from the original vertical position) so the cutter could pass through an empty space between them. The other stimuli and conditions were identical to Experiment 1.

#### Procedure

Participants performed the ball task for 10 minutes, and then remained standing in a T-pose and continued looking down until the end of the trial when the notification appeared on the HMD. After 10 seconds, the spinning cutter appeared and passed between the gloves and socks. Finally, they completed a questionnaire comprising twelve items on a seven-point Likert scale from −3 to 3.

##### Questionnaire

1. It felt as if the space between the gloves and socks in front of the mirror was my body.
2. It felt as if the space between the gloves and socks in the mirror was my body.
3. It felt as if the gloves in front of the mirror were part of my body.
4. It felt as if the gloves in the mirror were part of my body.
5. It felt as if the socks in front of the mirror were part of my body.
6. It felt as if the socks in the mirror were part of my body.
7. It seemed as if I could perceive a whole body between the gloves and socks in front of the mirror.
8. It seemed as if I could perceive a whole body between the gloves and socks in the mirror.
9. It felt as if I had been cut by the cutter.
10. The movement of the gloves and socks seemed to be my movement.
11. The movement of the gloves and socks seemed to be another person’s movement.
12. It seemed as if my body became a floor.

The first question concerned full body ownership (the second: in the mirror); the third and fourth, body part ownership (the fifth and sixth: in the mirror); the seventh, the perception of the invisible body by interpolating the hands and feet (the eighth: in the mirror); the ninth, the subjective startle response; and the tenth and eleventh, the degree of self-agency. The twelfth question was a control question to check random responses.

All participants performed eight experimental trials (within-subject design: two body conditions and two synchronisation conditions with two repetitions) in random order. Before the experimental trials, the participants touched 10 balls to practise the ball task in the normal layout and synchronous condition.

### Experiment 3

#### Participants

Twenty naïve and new volunteers (18 males and 2 females, mean age 22.2 ± 1.28 (*SD*)) participated in Experiment 3.

#### Apparatus, Stimuli, and Conditions

The apparatus, stimuli, and conditions were identical to Experiment 2.

#### Procedure

Participants performed the ball task for 12 minutes. In the last 2 minutes, the spinning cutter randomly appeared and passed between the gloves and socks three times.

All participants performed four experimental trials (within-subject design: two body conditions and two synchronisation conditions) in random order. Before the experimental trials, the participants touched 10 balls to practise the ball task in the normal layout and synchronous condition.

## Supplementary information

Supplementary Material
